# Impact of dengue virus infection on the cytoadherence of *Plasmodium vivax*-infected erythrocytes

**DOI:** 10.1590/0074-02760240185

**Published:** 2025-04-25

**Authors:** Maria Geuziane Soares da Cruz, Rafaella Oliveira dos Santos, Maria Gloria Teixeira Sousa, Fabio TM Costa, Marcus Vinícius Guimarães de Lacerda, Stefanie Costa Pinto Lopes, Pritesh Lalwani

**Affiliations:** 1 Fundação Oswaldo Cruz-Fiocruz, Instituto Leônidas e Maria Deane, Manaus, AM, Brasil; 2 Universidade Federal do Amazonas, Laboratório de Doenças Infeciosas e Imunologia, Manaus, AM, Brasil; 3 Universidade de São Paulo, Faculdade de Medicina, Instituto de Medicina Tropical, Hospital das Clínicas da Faculdade de Medicina da Universidade de São Paulo, Divisão de Dermatologia Clínica, Laboratório de Micologia Médica, São Paulo, SP, Brasil; 4 Universidade Estadual de Campinas, Departamento de Genética, Evolução, Bioagentes, Microbiologia e Imunologia, Laboratório de Doenças Tropicais, Campinas, SP, Brasil; 5 Fundação de Medicina Tropical Dr Heitor Vieira Dourado, Manaus, AM, Brasil

**Keywords:** Plasmodium, dengue virus, coinfection, cytoadherence, pathogenesis, severe disease

## Abstract

**BACKGROUND:**

Coinfections of *Plasmodium* parasites and the dengue virus have been linked to severe disease in some patients. The interactions between these two pathogens, particularly their effects on disease progression, highlight the clinical importance of understanding the mechanisms underlying the potential synergistic effects.

**OBJECTIVES:**

The primary objective of this study was to investigate the adhesion dynamics of *Plasmodium vivax*-infected erythrocytes (Pv-iRBCs) in the presence of dengue virus (DENV) infection. By examining the interaction between these pathogens, the study aimed to provide insights into how coinfections might influence disease severity and progression.

**METHODS:**

HepG2 cells were infected with DENV to observe changes in adhesion receptors and Pv-iRBCs adhesion capacity. Experiments using trypsin-treated Pv-iRBCs and UV-inactivated DENV dissected the adhesion process. Small molecule inhibitors were used to assess innate activation. ICAM-1 expression and its functional significance was quantified using a monoclonal anti-ICAM-1 antibody.

**FINDINGS:**

We noted a significant increase in cytoadherence of Pv-iRBCs following DENV infection compared to mock conditions. Both trypsin treatment of Pv-iRBCs and UV inactivation of DENV led to a reduction in cytoadherence, underscoring their impact on the adhesion process. Notably, DENV infection induces an innate immune activation upregulating ICAM-1 on the cell surface and blocking with a monoclonal anti-ICAM-1 antibody significantly reduced the cytoadherence of Pv-iRBCs.

**MAIN CONCLUSIONS:**

Elevated ICAM-1 levels on DENV-permissive cells may not only trap parasites within several niches but also contribute to endothelial and haematological disturbances in individuals with coinfections. Further research is required to fully elucidate the roles of cytoadherence and immune activation in the pathogenesis of dengue and malaria coinfections.

Dengue fever and malaria are two significant infectious diseases endemic to tropical regions, such as the Amazon, where they often co-circulate.^1^ ^,^ ^2^ Dengue fever is caused by the dengue virus (DENV), primarily transmitted by *Aedes* mosquitoes, particularly *Aedes aegypti*. In contrast, malaria is caused by *Plasmodium* parasites and transmitted by *Anopheles* mosquitoes. Coinfections with both DENV and *Plasmodium* parasites have been documented, leading to more severe symptoms and greater complications, posing challenges in diagnosis and concurrent management.

While *Plasmodium vivax* is considered less severe than *Plasmodium falciparum*, it presents unique challenges due to its ability to adhere to host cell surface proteins. This interaction can increase total parasite biomass in different tissues, which can activate endothelial cell and can cause haematological disturbances.^3^ By exploring the intricacies of cytoadhesion in *P. vivax*, particularly in the context of coinfection with DENV, this study aims to contribute to a broader understanding of malaria-dengue pathogenesis.

## SUBJECTS AND METHODS

*Patient samples* - A cross-sectional study design was used to recruit malaria patients at the Tropical Medicine Foundation (FMT-HVD) in Manaus, Amazonas between May, and August 2019. Symptomatic individuals over 18 years of age, with a positive microscopic thick smear for *P. vivax* with parasitaemia equal to or greater than two crosses, were included in the study, before starting any antimalarial treatment. After signing the informed consent, peripheral blood was collected in Heparin tubes. Patients with a microscopic diagnosis of *P. falciparum* or mixed infection (*P. vivax* and *P. falciparum*), pregnant women and patients who had recently used antimalarials such as chloroquine and primaquine were excluded from the study.

*Cell cultivation and maintenance* - HEP-G2 (ATCC HB-8065), A549 (ATCC CCL185), Vero (ATCC CCL81), and C6/36 (*Aedes albopictus* cell line, donated by Prof Dr Luiz Tadeu Figueiredo, FM-USP/Riberão Preto, São Paulo) cells were cultured under specific conditions. HEP-G2, A549, and Vero cells were grown in Dulbecco’s Modified Eagle Medium (DMEM) (Gibco), supplemented with 10% foetal bovine serum (FBS) (Gibco), 10,000 Units/mL of penicillin, and 10,000 µg/mL of streptomycin (Gibco), and maintained at 37**º**C with 5% CO₂. The C6/36 cells were cultured in Leibowitz L-15 (Gibco) medium supplemented with 10% FBS (Gibco), 10,000 Units/mL of penicillin, and 10,000 µg/mL of streptomycin (Gibco) and maintained at 28**º**C.

*Virus titration and infection* - DENV serotype 4 (Strain H241, donation of Prof Dr Luiz Tadeu Figueiredo, FM-USP/Riberão Preto, São Paulo) was propagated in C6/36 cells (*Ae. albopictus* cell line) at 28**º**C in Leibowitz L15 medium with 2% FBS. After six-seven days of infection, the supernatant was collected, centrifuged, and stored at -80**º**C for subsequent experiments. Virus titrations were performed on Vero cells. Cells were plated and incubated for 24 h. Next day, a 10-fold dilution of the viral stock was added for 1 h, and then a solution of 2X concentrated DMEM supplemented with 2% FBS and 1.5% carboxymethyl cellulose (CMC) was added. After five days, cells were fixed, permeabilised, and blocked. Primary antibodies from a serum pool of DENV-positive patients or 4G2 monoclonal antibody were added, followed by secondary anti-human or anti-mouse IgG antibodies conjugated with Horse radish peroxidase (HRP). TrueBlue substrate was used, and foci were counted visually to determine virus titre.

*Flow cytometry* - HepG2 and A549 cells were cultured until reaching 80% confluency. Upon trypsinisation, cells were resuspended in DMEM with 10% FBS. Fluorochrome-conjugated antibodies targeting adhesion molecules: PECAM-1 or CD31 (Clone WM59, 10 **μL**), ICAM-1 or CD54 (Clone HA58, 20 **μL**), and VCAM-1 or CD106 (Clone 5I-10C9, 20 **μL**) were added in a final volume of 50 **μL** with phosphate-buffered saline (PBS) + 10% foetal calf serum (FCS). All antibodies were APC-conjugated and Ig1k isotype (BD Biosciences and eBioscience). The cells were incubated for 30 min at 4**º**C. Following labelling, cells were washed three times with PBS and fixed with 0.5% paraformaldehyde-PBS. Cells were analysed on a FACSCanto II flow cytometer (Becton, Dickinson and Company, San Jose, CA, USA) and 20,000 events were recorded at the Leonidas and Maria Deane Institute (ILMD) - Fiocruz Amazon. FlowJo program (version 9.1) was employed for data analysis.

*Isolation of P. vivax-infected erythrocytes (Pv-iRBCs)* - Blood collected from the patient in a heparin tube (10 mL, BD Biosciences) underwent microscopic examination to confirm the presence of trophozoites and schizonts. Next, we depleted leukocytes and separated Pv-iRBCs with a density gradient centrifugation as previously described.^4^ Briefly, plasma was initially separated from whole blood by centrifuging at 2,500 rpm for 10 min at 4**º**C. Following plasma removal, the blood underwent a filtration process to eliminate white blood cells (WBCs) by passing it through a Whatman CF11 cellulose column. The resulting pellet was then washed three times with RPMI 1640 medium (Sigma, pH 7.2) to ensure purity, with each wash involving gentle resuspension and centrifugation to remove contaminants. After washing, the pellet was resuspended to achieve a 10% haematocrit.

To enrich for Pv-iRBCs containing mature parasite stages (trophozoites, schizonts, and gametocytes), a separation was performed to distinguish them from younger stages (rings) and uninfected erythrocytes. This involved layering 5 mL of the 10% erythrocyte suspension on top of 5 mL of 45% Percoll solution (Sigma P1644). The mixture was then centrifuged at 2,500 rpm for 20 min at 4**º**C, resulting in a clear interphase containing mature stages. This interphase layer was carefully collected and washed three times with RPMI 1640 medium (pH 7.2) to remove any residual Percoll.

To confirm the presence and concentration of mature Pv-iRBCs, thin blood smears were prepared and examined microscopically. The proportion of mature forms in the sample and the density of Pv-iRBCs per millilitre were quantified using a Neubauer chamber for precise cell counting (0.0025 mm² per chamber square).

*Cytoadhesion assay* - HepG2 cells, selected for their constitutive expression of the crucial adhesion molecule CD54 (ICAM-1), were utilised following a modified protocol.^4^ Upon reaching 80% confluence, cells were trypsinised and plated onto Lab-Tek 8-well slides. Cells were treated with varying multiplicity of infection (MOI)s for DENV, uvDENV (DENV inactivated by ultraviolet light), and inhibitors BX795 (10 **μM**/mL) or chloroquine (100 **μM**/mL). To investigate the influence of ICAM-1 in Pv-iE adhesion, cells were incubated with or without anti-CD54 (1:10, clone 15-2/ 84H10 Serotec AbD Catalog number MCA532). Additionally, trypsin-treated Pv-iRBCs were assessed for adhesion, exploring the role of parasite surface proteins. Serum pool of five patients with more than ten self-reported malaria infections was utilised to probe the potential blocking effect of antibodies. After treatment, 10^5^ Pv-iRBCs were added to each well and incubated for 1 h at 37**º**C. Post-incubation, non-adherent erythrocytes were meticulously washed away, and the remaining adhered Pv-iRBCs were quantified after fixing and staining with rapid panoptic.

*Data analysis* - Kruskal-Wallis test with Dunn’s multiple comparison test or two-way analysis of variance (ANOVA) with Tukey’s multiple comparisons test was performed to understand the differences between the study groups. All statistical analysis were performed using the GraphPad Prism software (v9.1.2 for Mac OS).

## RESULTS

**Fig. 1:**
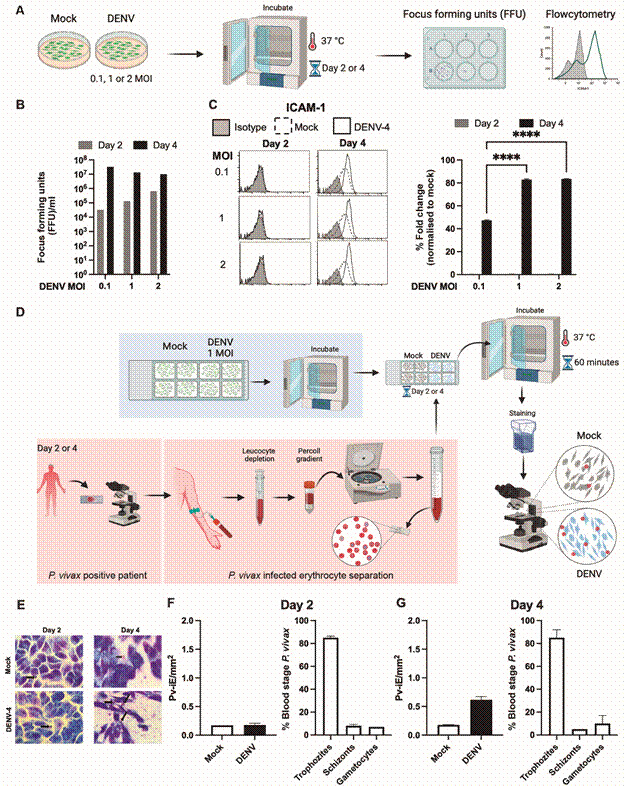
cells infected with dengue virus (DENV) upregulate ICAM-1, leading to increased cytoadherence of *Plasmodium vivax*-infected erythrocytes (Pv-iRBCs). HepG2 cells mock treated or infected with DENV at different multiplicity of infection (MOI). (A-B) Plaque assay was performed to measure DENV in cell supernatant, and (C) cells were stained with anti-ICAM-1 to measure cell surface expression. Representative histogram for ICAM-1 staining. Data are also expressed as percentage of control mean fluorescence intensity (MFI) and shown as mean +SD of six samples pooled from three experiments. (D) Schematic illustration depicts the experimental design. HepG2 cells were mock treated or infected with 1 MOI DENV and subsequently incubated with purified Pv-iRBCs. After incubation unbound parasite was washed and stained for microscopic counting. (E) Representative microscopic images of cytoadhered Pv-iRBCs. Adhered Pv-iRBCs were measured (F) Day 2 and (G) Day 4 after DENV infection. Adjacent graph demonstrates the percentage of parasite stages among the purified cells. Data is shown as mean +SD of one experiment performed with one donor in duplicate. Experiment was performed with five independent donors or five different isolates. ^****^ p < 0.0001; two-way analysis of variance (ANOVA) with Tukey’s multiple comparisons test.

The cytoadhesion process of *Plasmodium* parasites involves interactions between parasite-derived adhesion molecules and host cell receptors. Hence, we sought to investigate the expression of adhesion receptors on A549 and HepG2 cells. Notably, HepG2 cells exhibited a heightened expression of ICAM-1 on their cell surface compared to A549 cells. However, these HepG2 cells demonstrated low levels of PECAM-1 and did not express VCAM-1 proteins, as illustrated in Supplementary data (Fig. 1).

Next, we validated the permissiveness of HepG2 cells to DENV virus, as depicted in Fig. 1A-B. Additionally, upon DENV infection, we observed a notable increase in ICAM-1 expression on day 4 post-infection. This increase in ICAM-1 expression was particularly significant at 1 or 2 multiplicity of infection (MOI) compared to 0.1 MOI, as illustrated in Fig. 1C. Consequently, for all subsequent experiments, we adopted a consistent 1 MOI DENV infection protocol.

Subsequently, blood-stage *P. vivax* parasites were isolated from patient blood, and a cytoadhesion assay was conducted using cells that were either mock-treated or infected with DENV (Fig. 1D). The parasite preparations predominantly consisted of trophozoites, with a smaller percentage of schizonts and gametocytes. We noted a substantial augmentation in the cytoadhesion of *P. vivax* parasites on day 4 of DENV infection compared to mock-treated cells. This heightened adherence of the parasite correlated with an increase in ICAM-1 expression on day 4 post-infection (Fig. 1E-G).

**Fig. 2:**
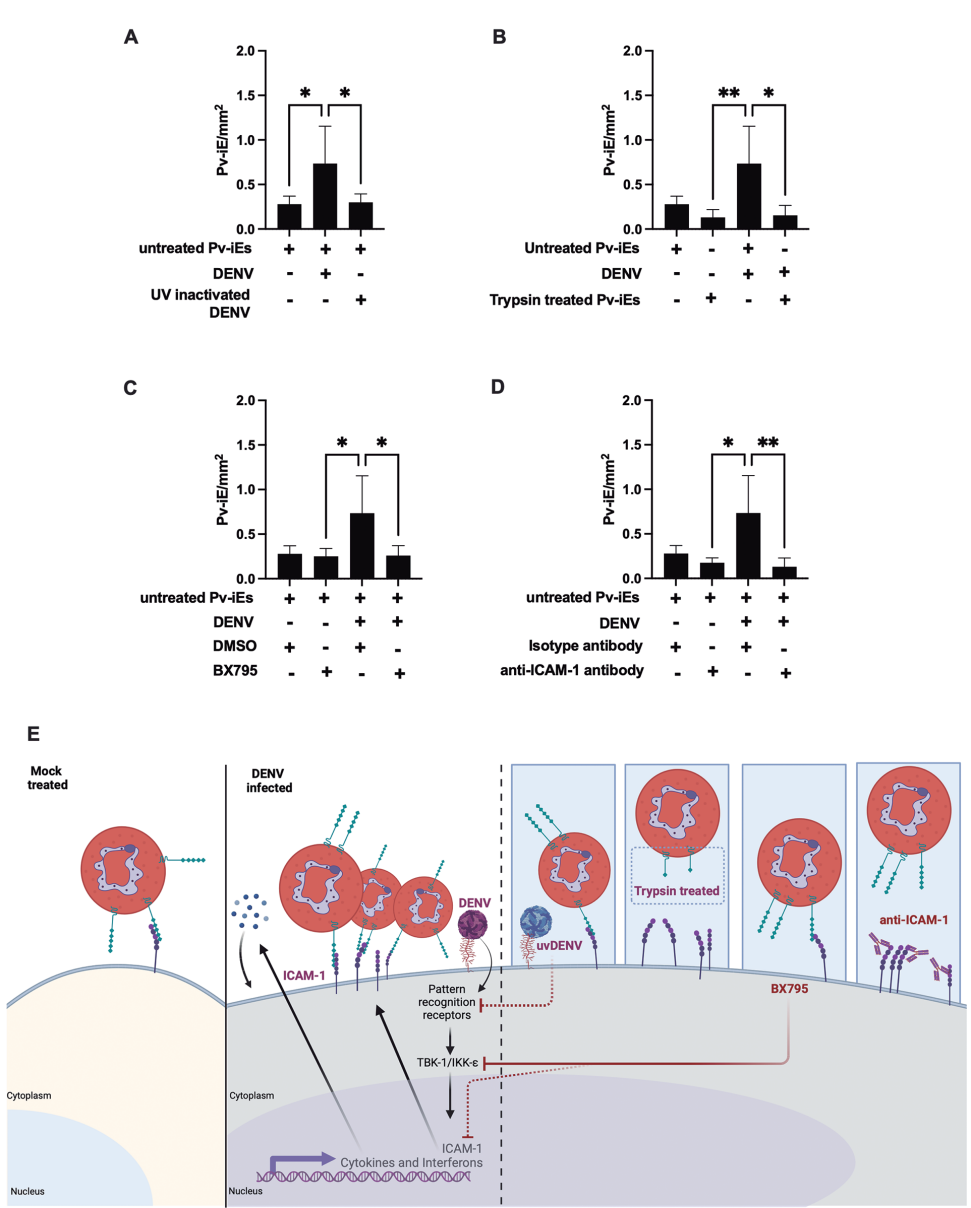
dengue virus (DENV) replication, innate activation and ICAM-1 essential for elevated Pv-iRBCs cytoadherence. HepG2 cells were infected with 1 multiplicity of infection (MOI) DENV and subsequently incubated with purified Pv-iRBCs. After incubation, unbound parasite was washed and stained for microscopic counting. (A) Cells were infected with live or UV-inactivated DENV. (B) Purified Pv-iRBCs were left untreated or treated with trypsin and then cytoadherence experiment was performed. (C) HepG2 cells were infected with DENV at 1 MOI in presence of vehicle-DMSO or BX795 small molecule to inhibit innate signalling and on day 4 cytoadhesion experiment was performed. (D) monoclonal anti-ICAM-1 antibody was added to mock treated or DENV infected cells, post incubation cytoadherence assay was performed with Pv-iRBCs. Data is shown as mean +SD of six samples pooled from three experiments, with three different isolates. ^*^ p < 0.05, ^**^ p < 0.01; Kruskal-Wallis test with Dunn’s multiple comparison test.

Next, we performed a cytoadhesion assay using UV-inactivated DENV, affirming the necessity for active virus replication in infected cells to augment parasite cytoadhesion (Fig. 2A). To further validate the role of parasite proteins expressed on Pv-iRBCs in cytoadherence, we treated Pv-iRBCs with trypsin, confirming the essential contribution of these proteins in parasite adherence (Fig. 2B).

Intriguingly, inhibition with BX795, a compound known to block the innate activation via TBK-1 signalling, abolished the observed increase in cytoadherence post DENV infection (Fig. 2C). However, inhibition with chloroquine, another inhibitor that blocks endosomal innate immune response, did not interfere with parasite cytoadherence [Supplementary data (Fig. 2)]. We also used pool of serum from *P. vivax* patients and controls to block the parasite-host cell receptor interaction, however, this pooled serum did not affect the Pv-iRBCs adherence [Supplementary data (Fig. 3)].

To underscore the significance of ICAM-1 in the cytoadherence of *P. vivax* parasites, we added an anti-ICAM-1 monoclonal antibody to inhibit this interaction. Inhibition or blocking with anti-ICAM-1 resulted in a reduced frequency of Pv-iRBCs adherence to both mock-treated and DENV infected cells (Fig. 2D). In summary, our findings indicate an elevation in ICAM-1 expression on the cell surface post DENV infection, leading subsequently to an increase in the cytoadherence of *P. vivax* parasites.

## DISCUSSION

In this study we demonstrate a substantial increase in cytoadherence of *P. vivax* infected erythrocytes to dengue infected cells *in vitro*. Notably, we demonstrate that this increase in adhesion was dependent of DENV replication, ICAM-1 expression on cell surface, and innate immune activation post DENV infection. Here we provide clues to the disease severity observed in coinfected individuals and provable mechanisms of pathogenesis that might be involved.

Human autopsies confirm parasite adherence to organs in various tissues,^5^ ^,^ ^6^ however, this cytoadhesive capacity is not exclusive to severe cases, as mature stages of the parasites are notably absent from the bloodstream of individuals with a mild clinical presentation. This observation underscores that the sequestration of mature forms in other tissues is an inherent mechanism within the biology of the parasite and is not solely associated with severe malaria cases.^7^ ^,^ ^8^ The understanding of cytoadhesion and sequestration of infected erythrocytes as an escape mechanism employed by the parasite against the host’s immune response, thereby preventing clearance in the spleen, is well-established.^9^ On the other hand, retention of this parasite biomass in tissues and organs positively correlates with increased endothelial cell activation and host response contributing to the immunopathogenesis.^3^

Trypsin-treated Pv-iRBCs couldn’t cytoadhere even with DENV infection, which confirms a physical binding between parasite proteins and cell receptors is essential. Failure of pool of serum from individuals with multiple *P. vivax* infection to block parasite and cell receptor interaction suggests significant antigenic differences between *P. vivax* isolates and/or adhesion domains might be antigenically restricted. Reinfection with *P. vivax* multiple times over a lifetime, despite previous exposures, underscores the challenge in achieving lasting immunity and highlights the complexity of correlates of protection against *P. vivax*.

There was no immediate increase in adhesion of infected erythrocytes or ICAM-1 expression after two days of DENV infection and ICAM-1 expression increased only on day 4. UV-inactivated DENV did not increase ICAM-1, emphasising the importance of virus replication. Blocking TBK-1 signalling confirmed the role of innate activation in upregulating ICAM-1^10^ and elevating Pv-iRBCs adhesion on DENV infected cells. Additionally, IFN-γ and MIF upregulates ICAM-1 through Erk, MAPK and PI3K signalling in DENV.^11^ However, blocking with chloroquine known to block endosomal pattern recognition receptor signalling did not influence the Pv-iRBCs adhesion. Moreover, blocking parasite and host cell receptor interaction with anti-ICAM-1 antibody, confirms its essential role in parasite accumulation in tissues and organs.

In this study, we could not perform double staining of virus-infected cells and parasite adhered to the cells due to technical constraints. Here, we performed experiments with DENV4 and expect similar response with other DENV serotypes since ICAM-1 induction after DENV infection has been described with different serotypes. Here we observed low level of Pv-iRBCs adhesion compared to previously reported for endothelial cells; variation in expression of ICAM-1 and additional adhesion receptors like PECAM-1 and VCAM-1 could have influenced the frequency of Pv-iRBCs adhering to the cells. Here we tested epithelial cells, but DENV permissive leucocytes, endothelial and bone marrow cells that express ICAM-1 or other adhesion receptors would invariably participate in parasite-mass retention. Besides, we conclusively demonstrate an increase in cytoadherence after DENV infection was dependent on ICAM-1 expression, innate activation and DENV replication. These results provide new insight into DENV-malaria coinfection. The ubiquitous expression of ICAM-1 in several virus permissive cell types (https://www.proteinatlas.org/ENSG00000090339-ICAM1) can be modulated during virus replication; additionally, *Plasmodium* sequestration in various organs may not only stimulate immune responses but also influence parasitaemia and transmission. Further studies are needed to determine whether other endemic viral infections also modulate surface adhesion receptors in these cell types, potentially increasing parasite sequestration during malaria coinfections. However, further studies in vivo with DENV-malaria coinfection can shed light on the role of this cytoadherence and immune activation in disease pathogenesis. This study also alerts us to greater attention towards patients diagnosed with dengue or malaria mono-infection in endemic areas with parasite and virus co-circulation.
