# Supplementary material for: Impact of dengue virus infection on the cytoadherence of Plasmodium vivax-infected erythrocytes
Source: Mem Inst Oswaldo Cruz. 2025 Apr 25;120:e240185. doi: 10.1590/0074-02760240185 (PMC12039924; doi:10.1590/0074-02760240185)
Supplement: Supplementary file 1 [file 1678-8060-mioc-120-e240185-s.pdf]

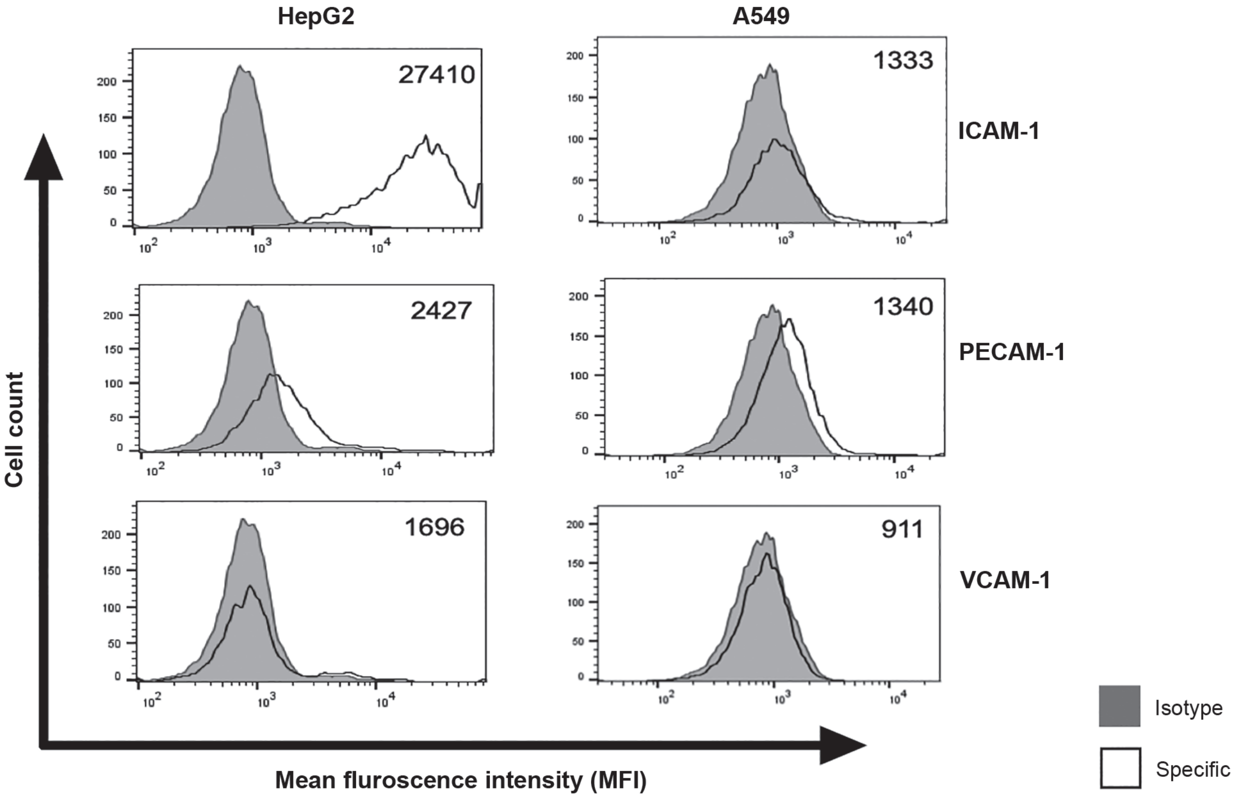

Fig. 1: expression of adhesion molecules on HepG2 and A549 cells. Uninfected cells were stained to check constitutive surface expression of adhesion molecules by flowcytometry.

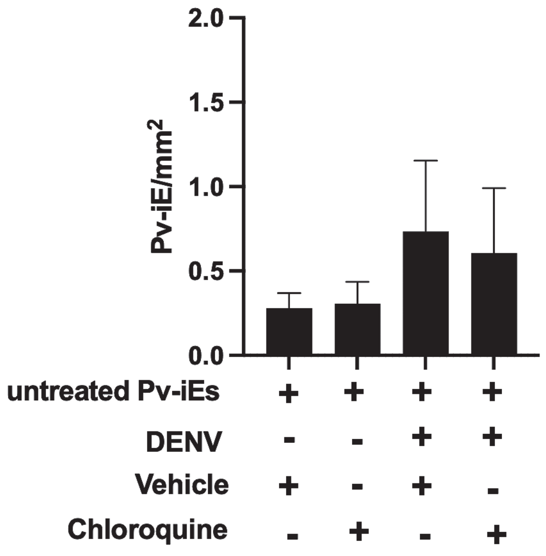

Fig. 2: Pv-iRBCs cytoadherence in presence of *Plasmodium vivax* patient serum. HepG2 cells were infected with 1 multiplicity of infection (MOI) dengue virus (DENV) and subsequently incubated with purified Pv-iRBCs. After incubation, unbound parasite was washed and stained for microscopic counting. Pv-iRBCs were incubated with serum pool of *P. vivax* patients or treated with *P. vivax* negative serum pool, after incubation cytoadherence assay was performed with Pv-iRBCs. Data is shown as mean +SD of six samples pooled from three experiments.

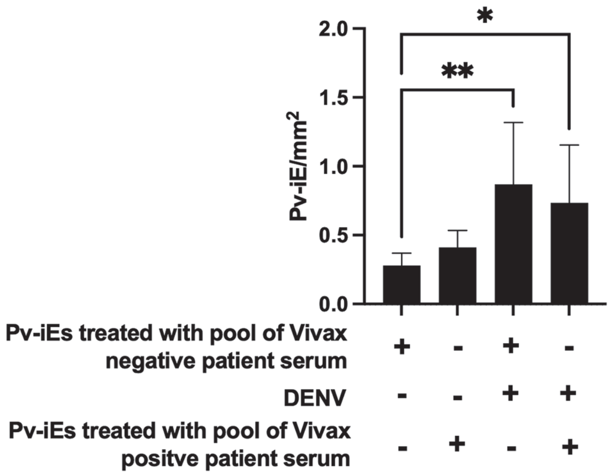

Fig. 3: inhibition with chloroquine does not alter Pv-iRBCs cytoadherence. HepG2 cells were infected with dengue virus (DENV) at 1 multiplicity of infection (MOI) in presence of vehicle or chloroquine small molecule to inhibit innate signalling and on day 4 cytoadhesion experiment was performed. Data is shown as mean +SD of six samples pooled from three experiments. $^{*}p < 0.05$, $^{**}p < 0.01$; Kruskal-Wallis test with Dunn's multiple comparison test.

CC BY
